# Contrasting Toxicity Classes Differentially Affect Gut Microbiota Composition in Honey Bees

**DOI:** 10.3390/insects17040437

**Published:** 2026-04-20

**Authors:** Yunchao Kan, Ruoke Wang, Bing Zhang, Yu Liu, Runqiang Liu, Zhongyin Zhang, Zhaonan Zhang, Camilo Ayra-Pardo, Dandan Li

**Affiliations:** 1 School of Plant Protection and Environment, Henan Institute of Science and Technology, 90 East of Hualan Avenue, Xinxiang 453003, China; kanyunchao@163.com; 2 Henan Key Laboratory of Insect Biology, The International Joint Laboratory of Insect Biology in Henan Province, Nanyang Normal University, 1638 Wolong Road, Nanyang 473061, China; 15028842796@163.com (R.W.); zb964034798@163.com (B.Z.); e20022096@163.com (Y.L.); liurunqiang1983@163.com (R.L.); zzy206@126.com (Z.Z.); 3 College of Bee Science and Biomedicine, Fujian Agriculture and Forestry University, Fuzhou 350001, China; 000Q825148@fafu.edu.cn; 4 CIIMAR/CIMAR LA, Interdisciplinary Centre of Marine and Environmental Research, University of Porto, Terminal de Cruzeiros do Porto de Leixões, Avda. General Norton de Matos s/n, 4450-208 Matosinhos, Portugal

**Keywords:** *Apis mellifera*, pesticide risk assessment, 16S rRNA gene sequencing

## Abstract

Honey bees rely on a specialized gut microbiota for nutrition, detoxification, and immune function, yet the effects of emerging insecticides on this symbiotic system remain poorly understood. We compared the acute toxicity and short-term gut microbiota responses of *Apis mellifera ligustica* workers exposed to two insecticides with contrasting toxicity classes: the highly toxic emamectin benzoate-lufenuron (EB-LFR) and the low-toxicity ecdysone agonist RH-5849. EB-LFR was associated with observed reductions in core gut symbionts (*Gilliamella*, *Snodgrassella*, *Lactobacillus*), a transient increase in *Bifidobacterium*, and the detection of opportunistic taxa such as *Serratia marcescens* and *Enterobacter hormaechei*. In contrast, RH-5849 was associated with broad reductions in beneficial bacteria without detectable pathogen emergence, suggesting a more moderate alteration of microbiota composition. Because microbiota analyses were based on single pooled samples per treatment, these results represent exploratory, qualitative insights into early microbial responses. Together with acute toxicity data, the findings suggest that insecticides with contrasting toxicity classes may differentially affect gut microbiota composition in honey bees and highlight the value of incorporating gut microbiota endpoints into pesticide risk-assessment frameworks to better anticipate sublethal effects on pollinator health.

## Simple Summary

Honey bees depend on a small but highly specialized community of gut bacteria that help them digest food, resist infections, and cope with environmental stress. Many newer insecticides are promoted as “reduced-risk,” yet their effects on this microbial system remain poorly understood. In this study, we compared how two insecticides with contrasting toxicity levels—emamectin benzoate-lufenuron (high toxicity) and RH-5849 (low toxicity)—affect honey bee survival and gut microbiota composition. The highly toxic insecticide was associated with observed reductions in beneficial bacteria and the detection of opportunistic microbes, whereas the low-toxicity insecticide was associated with a broader depletion of beneficial taxa without apparent pathogen emergence. Because the microbiota results were based on pooled samples, these observations should be considered exploratory. Nevertheless, they suggest that even insecticides considered relatively safe may influence the gut microbial composition. Evaluating microbial responses alongside traditional toxicity tests may help improve the protection of pollinators.

## 1. Introduction

Honey bees (*Apis mellifera*) are essential pollinators in natural and agricultural ecosystems, supporting global food security and biodiversity through their contributions to fruit set, seed production, and crop yield [1]. Despite their ecological and economic importance, honey bee populations have declined markedly in recent decades [2,3]. In the United States, managed colonies decreased from six million in 1947 to 2.5 million in 2022, with annual losses averaging 30% [4]. Similar declines are reported in Asia, where the distribution of the Eastern honey bee (*A. cerana cerana*) has contracted by 60% due to habitat degradation and biological invasions [5]. These losses arise from multiple interacting stressors, including parasites, pathogens, nutritional deficits, climate change, and pesticide exposure [6].

Among abiotic stressors, insecticides remain one of the most pervasive threats to honey bee health. Neonicotinoids such as imidacloprid and thiamethoxam impair navigation, learning, and foraging efficiency, ultimately reducing colony survival [7,8]. Pyrethroids disrupt voltage-gated sodium channels, causing motor dysfunction and dramatically reducing homing success [9]. In response to these risks, regulatory agencies have restricted or banned several neurotoxic insecticides, prompting a shift toward alternative chemistries with lower perceived toxicity. However, the ecological safety of many newer compounds remains insufficiently characterized, particularly regarding their sublethal effects on bee physiology and gut microbial homeostasis.

Emamectin benzoate–lufenuron (EB-LFR) is a co-formulated insecticide widely used to control lepidopteran pests in fruit trees, vegetables, and cotton [10,11]. Emamectin benzoate acts on glutamate-gated chloride channels, while lufenuron inhibits chitin synthesis, jointly exerting potent stomach and contact toxicity. Both active ingredients have documented adverse effects on non-target organisms, including oxidative stress, reproductive impairment, and developmental disruption in aquatic and terrestrial species [12,13]. Although previous work has demonstrated high acute toxicity of this formulation to honey bees [14], its sublethal impacts—particularly on gut microbial stability—remain unexplored. RH-5849 (1,2-dibenzoyl-1-(t-butyl) hydrazine) is a nonsteroidal ecdysone agonist and insect growth regulator that suppresses larval feeding and disrupts molting [15,16]. It is considered a reduced-risk insecticide due to its slow action, low mammalian toxicity, and limited environmental persistence. Studies in other insects indicate that ecdysone agonists generally exhibit low toxicity to beneficial arthropods and minimal disruption of non-target physiological processes [17,18]. Despite its increasing use as an alternative to neurotoxic insecticides, no published studies have evaluated its toxicity or microbiota-level effects in honey bees, and existing work has focused primarily on receptor-level characterization rather than whole-organism responses [19]. EB-LFR is the highly toxic insecticde for honey bees (LD50 < 2 μg a.i./bee), whereas RH-5849 is consistent with the low-toxicity range (LD50 > 11 μg a.i./bee), thereby providing a clear framework for comparative analysis of insecticides with contrasting toxicity profiles. Understanding how such compounds influence bee gut symbiosis is therefore critical, as sublethal disturbances may compromise immunity, nutrition, and pathogen resistance even in the absence of acute mortality.

The honey bee gut microbiota is a simple but highly specialized community dominated by 8–10 core bacterial phylotypes [20,21]. Five taxa, including *Gilliamella apicola*, *Snodgrassella alvi*, *Bifidobacterium asteroides*, *Lactobacillus Firm-4*, and *Lactobacillus Firm-5*, form a conserved symbiotic consortium essential for digestion, detoxification, immune modulation, and pathogen defense [22,23,24,25,26,27,28,29,30]. This community exhibits functional resilience under moderate environmental stress, but severe perturbations can disrupt colonization dynamics, metabolic interactions, and immune signaling [31,32]. Dysbiosis, characterized by loss of core taxa, opportunistic pathogen expansion, or altered metabolic function, has been linked to increased pathogen susceptibility, impaired nutrient assimilation, and reduced host survival [28,29,33,34,35,36]. Pesticide exposure is a major driver of dysbiosis in honey bees. Neonicotinoids reduce the abundance of *Gilliamella*, *Lactobacillus*, and *Bifidobacterium*, while pyrethroids alter the relative abundance of *Bartonella*, *Serratia*, and *Frischella* [37,38,39,40]. However, most studies focus on single compounds [32,41], and comparative analyses of how high- versus low-toxicity insecticides differentially affect gut microbiota structure and function remain scarce.

This study addresses this knowledge gap by comparing the acute toxicity and short-term gut microbiota responses of honey bees exposed to two insecticides with contrasting toxicity profiles: the highly toxic EB-LFR and the low-toxicity ecdysone agonist RH-5849. We determined oral and contact LD_50_ values and characterized microbiota composition at 24 h and 48 h post-exposure using 16S rRNA gene sequencing. Our results provide exploratory, qualitative insights into how insecticides with contrasting toxicity classes may differentially affect gut microbiota composition in honey bees. These findings highlight the importance of incorporating gut microbiota endpoints as complementary measures in pesticide risk assessment frameworks.

## 2. Materials and Methods

### 2.1. Insects

Honey bees (*Apis mellifera ligustica*) were obtained from 6 healthy colonies maintained at the Henan Institute of Science and Technology (Xinxiang, Henan, China). Workers were randomly sampled from colonies to minimize colony-specific bias. Colonies were routinely inspected and confirmed to be free of Varroa destructor and *Nosema* spp., and no acaricides or antibiotics had been applied for at least three months prior to sampling. Newly emerged workers were marked and reared under natural colony conditions for 12 days for toxicity assays. Prior to experimentation, bees were acclimated in the laboratory and provided with 50% (*w*/*v*) sucrose solution ad libitum for 24 h, followed by a 2 h starvation period. All procedures complied with institutional guidelines for the ethical use of invertebrates in research.

### 2.2. Acute Oral Toxicity Assay

Acute oral toxicity was evaluated following standard protocols. EB-LFR was diluted in 50% sucrose solution to six nominal concentrations (0.2, 0.4, 0.6, 0.8, 1.0, and 1.2 mg/L). RH-5849 was dissolved in acetone to prepare a stock solution and subsequently diluted with sucrose solution to five concentrations (250, 500, 750, 1000, and 1250 mg/L), resulting in a low final acetone concentration. An acetone-only control was not included.

For each concentration, 500 μL of test solution was dispensed into a Petri dish placed inside a ventilated test cage. Bees were briefly immobilized at −20 °C for 2 min and transferred to cages, where they were allowed to feed freely. A control group received sucrose solution only. All bees were maintained at 25 ± 2 °C, 50–70% relative humidity, and a 14:10 h light:dark photoperiod in a programmable environmental incubator PGX-450A (Ningbo Prandt Instrument Co., Ltd., Ningbo, Zhejiang, China). Each treatment consisted of three replicate groups of 20 bees. Bees were randomly assigned to treatments, and mortality assessment was performed blind to treatment identity. Mortality was recorded at regular intervals up to 72 h. Oral exposure was defined by the concentration of the test solution provided under group-feeding conditions, without assuming a fixed per-bee consumption volume.

### 2.3. Acute Contact Toxicity Assay

Contact toxicity was assessed for EB-LFR. The pesticide was diluted in 50% sucrose solution to six concentrations (4, 6, 8, 10, 12, and 16 mg/L). Bees were immobilized by cold anesthesia, and a 1 μL droplet of test solution was applied to the dorsal thorax using a calibrated microapplicator (Eppendorf, Hamburg, Germany). Control bees received 1 μL of water. Treated bees were maintained under the same environmental conditions as in the oral assay. Each concentration included three replicate groups of 15 bees. Mortality was recorded immediately after treatment and at subsequent time points up to 72 h. Dimethoate was used as a reference toxicant in both oral and contact assays following identical procedures. Contact toxicity was not evaluated for RH-5849, as the experimental design prioritized oral exposure, which represents the primary route of activity for ecdysone agonists in insects.

### 2.4. Gut Dissection, DNA Extraction, and 16S rRNA Gene Sequencing

Although bees were reared to 12 days of age for toxicity assays, 7-day-old workers were selected for microbiota analysis because the core gut microbiota is typically established within approximately 5–7 days after adult emergence [42]. Bees were exposed to concentrations corresponding to the 48 h oral LD_50_ of EB-LFR or RH-5849. Surviving bees were collected at 24 h and 48 h post-exposure, anesthetized on ice, and surface-sterilized with 70% ethanol. The midgut and hindgut were dissected under sterile conditions.

Because individual honey bee guts yield limited microbial biomass, guts from ten workers were pooled to generate a single composite sample for each treatment and time point. Due to logistical constraints, only one pooled sample per group was available for sequencing; therefore, analyses requiring biological replication (e.g., statistical comparisons of alpha or beta diversity) were not performed.

Pooled gut samples were flash-frozen in liquid nitrogen and stored at −80 °C until processing. Total microbial DNA was extracted using the MagPure Stool DNA KF Kit B (MAGEN, Guangzhou, China). The V3-V4 region of the 16S rRNA gene was amplified using primers 338F (ACTCCTACGGGAGGCAGCAG) and 806R (GGACTACHVGGGTWTCTAAT). Amplicons were purified with DNA magnetic beads (BGI, LB00V60), and library quality was assessed using an Agilent 2100 Bioanalyzer (Agilent Technologies, Santa Clara, CA, USA). Sequencing was performed on the DNBSEQ-G400 platform (BGI Technologies. Co., Ltd., Shenzhen, China).

Raw reads were demultiplexed and trimmed using Cutadapt v2.6. Quality filtering employed a sliding-window approach (window size 30 bp; mean quality threshold Q20). Reads containing ambiguous bases, low complexity (≥10 identical consecutive bases), or <75% of original length after trimming were removed. Paired-end reads were merged using FLASH v1.2.11 (minimum overlap 15 bp; mismatch rate 0.1). For OTU-based analysis, merged tags were clustered at 97% similarity using USEARCH v11.0.667, and chimeras were removed using UCHIME v4.2.40. For ASV-based analysis, denoising was performed using DADA2 implemented in QIIME2 v2023.7. Taxonomic assignment was conducted using the RDP Classifier v1.9.1 (confidence threshold 0.6).

Samples yielded an average of 52,000 reads (range: 41,000–63,000). Samples with fewer than 20,000 reads were excluded. Taxa with mean relative abundance < 0.5% or unclassified at higher ranks were grouped as “Others.” Because only one pooled sample per treatment was available, microbiota results are presented descriptively, focusing on relative abundance patterns rather than statistical inference. Both OTU- and ASV-based analyses yielded comparable qualitative taxonomic profiles within samples; therefore, results are presented using OTU-based relative abundance profiles for clarity.

### 2.5. Statistical Analysis

Mortality data were analyzed using SPSS 27.0 (IBM Corp., Armonk, NY, USA). Mortality percentages were transformed to probit units, and concentrations were log-transformed. Median lethal doses (LD_50_) and 95% confidence intervals (95% CI) were estimated using probit regression. Data were tested for normality and homogeneity of variances prior to analysis. Differences among treatments were evaluated using one-way ANOVA, and when significant, Duncan’s multiple range test was applied (*p* < 0.05). Graphs were generated using GraphPad Prism 10.0 (GraphPad Software, LLC, San Diego, CA, USA).

## 3. Results

### 3.1. Acute Oral and Contact Toxicity of EB-LFR

Dimethoate was used as a reference toxicant to validate assay performance. The 24 h contact LD_50_ for *A. mellifera ligustica* was 0.128 μg active ingredient (a.i.)/bee (95% CI: 0.103–0.161 μg a.i./bee), and the 24 h oral LD_50_ was 0.123 μg a.i./bee (95% CI: 0.105–0.144 μg a.i./bee), values consistent with established sensitivity ranges. For oral exposure, dose values expressed as μg a.i./bee represent estimated values derived from group-feeding conditions.

**Figure 1:**
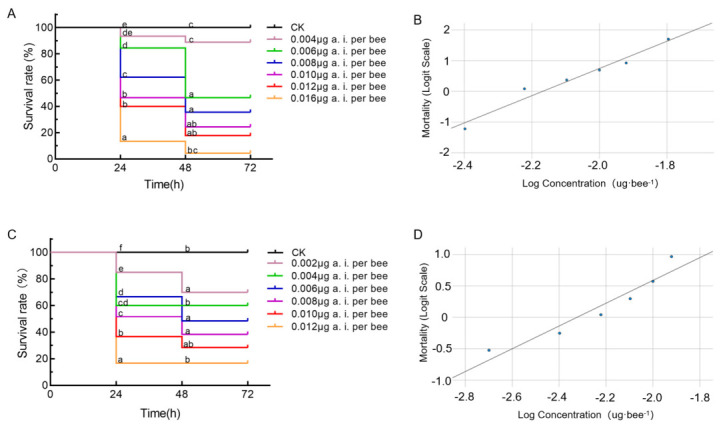
Survival and dose-mortality responses of adult honey bees exposed to emamectin benzoate-lufenuron (EB-LFR) through contact and oral exposure. (**A**) Survival dynamics of adult workers treated by topical application on the tergum over 72 h. Each treatment included three replicates of 15 bees, and data are presented as mean ± SEM. Distinct letters indicate statistically significant differences among treatments (*p* < 0.05; one-way ANOVA followed by Duncan’s test). (**B**) Dose-mortality response for topical exposure, showing logit-transformed mortality as a function of log-transformed concentration (μg a.i./bee). The fitted logistic regression is logit(mortality) = 9.62 + 4.43 × log(concentration). (**C**) Survival dynamics of adult workers exposed through feeding over 72 h, with three replicates of 20 bees per treatment. Data are presented as mean ± SEM, and distinct letters indicate statistically significant differences among treatments (*p* < 0.05; one-way ANOVA followed by Duncan’s test). (**D**) Dose-mortality response for oral exposure, showing logit-transformed mortality relative to log-transformed estimated dose (μg a.i./bee). The fitted logistic regression is logit(mortality) = 4.22 + 1.81 × log(concentration).

In the contact toxicity assay with EB-LFR, bees received 1 μL droplets delivering 0.004–0.016 μg a.i./bee, and survival declined in a clear dose-dependent manner. Mortality continued to increase between 24 h and 48 h at higher doses, indicating delayed toxic effects (Figure 1A). One-way ANOVA revealed significant differences among treatments at 24 h (*F*_6,14_ = 71.033, *p* < 0.0001) and 48 h (*F*_6,14_ = 8.131, *p* = 0.00064). Consistent with this trend, the 48 h contact LD_50_ estimated from the fitted logit regression was 0.0067 μg a.i./bee (95% CI: 0.0060–0.0073 μg a.i./bee) (Figure 1B).

Under oral exposure, estimated doses ranged from 0.002 to 0.012 μg a.i./bee, and survival similarly declined with increasing dose. In contrast to contact exposure, mortality differences between 24 h and 48 h remained below 10% across treatments, indicating limited delayed effects (Figure 1C). One-way ANOVA also showed significant differences among treatments at 24 h (*F*_6,14_ = 43.457, *p* < 0.0001) and 48 h (*F*_6,14_ = 6.583, *p* = 0.0018). The corresponding 48 h oral LD_50_ estimated from the fitted logit regression was 0.0048 μg a.i./bee (95% CI: 0.0039–0.0057 μg a.i./bee) (Figure 1D). According to GB/T 31270.10-2014, EB-LFR is classified as highly toxic to honey bees (LD_50_ between 0.001 and 2.0 μg a.i./bee).

### 3.2. Effects of EB-LFR on Gut Microbiota Composition

Sequencing of pooled gut samples revealed changes in community composition following exposure to EB-LFR. Because microbiota data are based on single pooled samples without biological replication, results are presented descriptively and interpreted qualitatively.

#### 3.2.1. 24 h Post-Exposure

**Figure 2:**
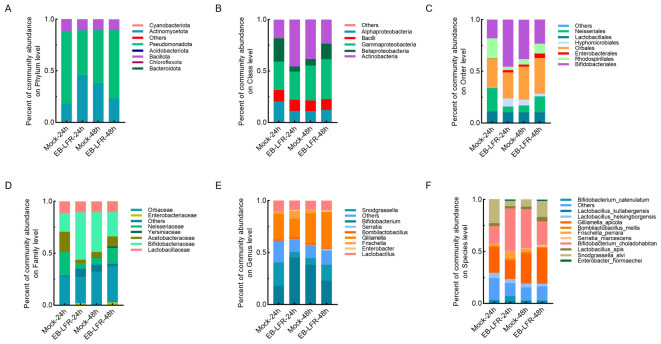
Effects of emamectin benzoate-lufenuron (EB-LFR) on the composition of the honey bee gut microbiota at 24 h and 48 h post-exposure. Panels show relative abundances at the (**A**) phylum, (**B**) class, (**C**) order, (**D**) family, (**E**) genus, and (**F**) species levels. Each bar represents a single pooled sample per treatment and time point. Mock refers to a 50% sucrose solution without EB-LFR. Data are derived from a single pooled sample per treatment and time point; therefore, results are presented as descriptive relative abundance profiles and interpreted qualitatively.

At 24 h, the pooled sample exposed to EB-LFR showed changes in gut microbiota composition. At the phylum level, Actinomycetota increased, whereas Pseudomonadota declined (Figure 2A). At the class level, Actinobacteria were enriched, while Alphaproteobacteria and Betaproteobacteria were reduced (Figure 2B). These shifts were reflected at finer taxonomic scales: Bifidobacteriales and Enterobacterales became more prominent, whereas Neisseriales and Rhodospirillales decreased (Figure 2C). Correspondingly, Bifidobacteriaceae and Enterobacteriaceae increased in relative abundance, while Neisseriaceae and Acetobacteraceae declined (Figure 2D).

At the genus level, *Bifidobacterium* showed an increase in relative abundance, whereas the core symbionts *Gilliamella* and *Snodgrassella* were reduced (Figure 2E). Species-level patterns showed declines in *Snodgrassella alvi* and *Gilliamella apicola*, accompanied by increases in *Bifidobacterium choladohabitans* and *Bombilactobacillus mellis*. Opportunistic taxa, including *S. marcescens* and *E. hormaechei*, were detected following exposure (Figure 2F).

#### 3.2.2. 48 h Post-Exposure

By 48 h, the microbiota composition shifted again. Actinomycetota decreased relative to 24 h, while Pseudomonadota increased (Figure 2A). At the class level, Gammaproteobacteria and Betaproteobacteria became more abundant (Figure 2B). Bifidobacteriales declined compared with 24 h, whereas Neisseriales and Acetobacterales increased (Figure 2C). Despite these changes, Bifidobacteriaceae and Enterobacteriaceae remained elevated relative to the control, while Neisseriaceae and Acetobacteraceae continued to show reduced abundance (Figure 2D).

At the genus level, *Bifidobacterium* decreased compared with 24 h, while *Gilliamella* and *Snodgrassella* showed partial recovery (Figure 2E). Species-level data indicated increases in *Snodgrassella alvi* and *Lactobacillus apis*, a decline in *Bifidobacterium choladohabitans*, and persistent detection of *Enterobacter hormaechei* (Figure 2F). Overall, EB-LFR was associated with changes in microbiota composition, including reductions in core symbionts, detection of opportunistic taxa, and partial recovery by 48 h.

### 3.3. Oral Toxicity of RH-5849

**Figure 3:**
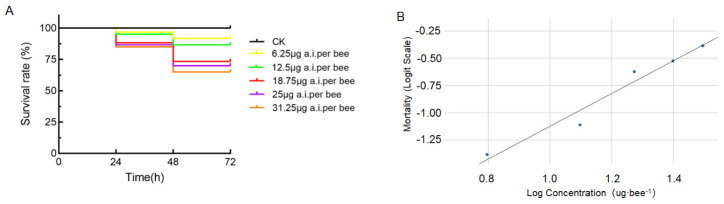
Survival and dose-mortality responses of adult honey bees exposed to RH-5849 through feeding. (**A**) Survival dynamics over 72 h for bees receiving different doses of RH-5849. Each treatment included three replicates of 20 adult bees, and data are presented as mean ± SEM. No statistically significant differences among treatments were detected (one-way ANOVA, *p* > 0.05). (**B**) Dose-mortality response showing logit-transformed mortality as a function of log-transformed estimated dose (μg a.i./bee). The fitted logistic regression is described by the equation: logit(mortality) = −2.63 + 1.50 × log(concentration).

RH-5849 exhibited low acute toxicity to adult honey bees. Across the tested concentration range (250–1250 mg/L), no overt poisoning symptoms were observed within the first 6 h of exposure. Survival declined gradually in a dose-dependent manner over 72 h (Figure 3A). Dose values (μg a.i./bee) are estimated from group-feeding conditions and increased with concentration. One-way ANOVA did not detect significant differences among treatments at 24 h (*F*_6,14_ = 1.987, *p* = 0.136) or 48 h (*F*_6,14_ = 2.472, *p* = 0.076), indicating limited statistical separation among dose groups. The fitted logit regression, logit(mortality) = −2.63 + 1.50 × log(concentration), yielded an estimated 48 h LD_50_ of 54.96 μg a.i./bee (95% CI: 36.55–159.18 μg a.i./bee) (Figure 3B). This value exceeds the 11 μg a.i./bee threshold for low-toxicity classification (GB/T 31270.10-2014), confirming RH-5849 as a low-toxicity insecticide.

### 3.4. Effects of RH-5849 on Gut Microbiota Composition

Sequencing of pooled gut samples revealed shifts in community composition following exposure to RH-5849. Because only one pooled sample per treatment was analyzed, microbiota results are presented as exploratory and descriptive patterns without statistical inference.

**Figure 4:**
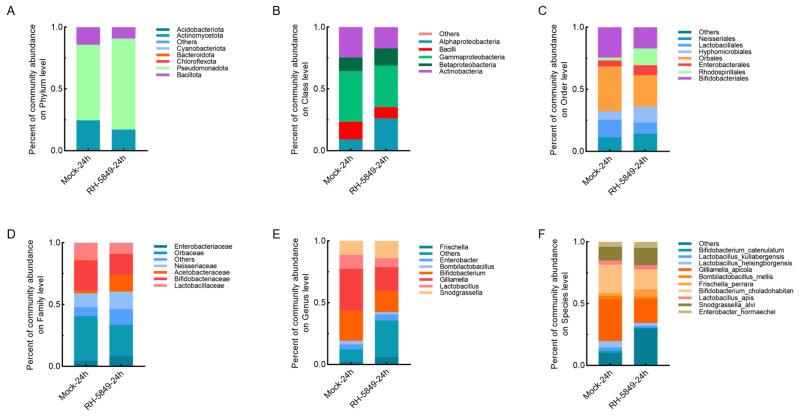
Effects of RH-5849 on the composition of the honey bee gut microbiota. Panels show relative abundances at the (**A**) phylum, (**B**) class, (**C**) order, (**D**) family, (**E**) genus, and (**F**) species levels. Each bar represents a single pooled sample per treatment. Mock refers to a 50% sucrose solution. Data are derived from a single pooled sample per treatment and time point; therefore, results are presented as descriptive relative abundance profiles and interpreted qualitatively.

Following exposure to RH-5849, changes in microbiota composition were observed across multiple taxonomic levels (Figure 4). At the phylum level, Actinomycetota and Bacteroidota decreased in relative abundance, whereas Pseudomonadota increased (Figure 4A). This pattern was reflected at lower taxonomic levels, with increases in Alphaproteobacteria and Betaproteobacteria and reductions in Bacilli and Actinobacteria (Figure 4B). Consistent with these shifts, orders associated with core symbionts, including Bifidobacteriales, Lactobacillales, and Orbales, showed reduced representation, while Rhodospirillales and Hyphomicrobiales increased (Figure 4C).

At the family level, Bifidobacteriaceae, Lactobacillaceae, Enterobacteriaceae, and Orbaceae were reduced, whereas Acetobacteraceae increased (Figure 4D). At the genus level, *Bifidobacterium*, *Gilliamella*, and *Lactobacillus* showed decreased relative abundance, while *Frischella* and *Snodgrassella* increased (Figure 4E). These trends were consistent at the species level, with reductions in *Bifidobacterium choladohabitan*, *Gilliamella apicola*, and *Lactobacillus helsingborgensis*, accompanied by an increase in *Frischella perrara* (Figure 4F). In contrast to EB-LFR, no opportunistic taxa such as *S. marcescens* or *E. hormaechei* were detected following RH-5849 exposure. Instead, the observed changes were characterized by a broad reduction in core symbionts without the emergence of detectable opportunistic bacteria.

### 3.5. Comparative Summary of Microbiota Responses

A qualitative comparison of the pooled samples revealed differences in observed patterns of gut microbiota alteration in honey bees exposed to insecticides with contrasting toxicity profiles. EB-LFR exposure showed reductions in core symbionts, transient increases in certain taxa, and the detection of opportunistic bacteria. In contrast, RH-5849 exposure showed a general reduction in beneficial bacteria without detectable pathogen emergence, consistent with a more moderate alteration of microbiota composition. Because the microbiota analysis was based on single pooled samples, these patterns should be interpreted as exploratory and hypothesis-generating, providing a basis for future replicated studies.

## 4. Discussion

The present study suggests that insecticides with contrasting toxicity classes are associated with differences in gut microbiota response patterns in honey bees. Although the microbiota results are based on single pooled samples and are therefore exploratory, the observed patterns are consistent with the possibility that toxicity class may influence the stability and composition of the honey bee gut microbiota. EB-LFR, a highly toxic formulation, was associated with reductions in core symbionts and the detection of opportunistic taxa, whereas RH-5849, a low-toxicity ecdysone agonist, was associated with a broader reduction in beneficial bacteria without apparent pathogen emergence. These observations are consistent with previous work showing that pesticide chemistry and mode of action can influence microbial homeostasis in bees [43,44,45,46,47,48,49], and they highlight the importance of considering sublethal microbial responses alongside mortality in pesticide risk assessment. Although group-feeding assays are standard for oral toxicity testing in honey bees, they inherently limit the precision of per-bee dose estimates. This does not affect toxicity classification but should be considered when comparing absolute LD_50_ values across studies.

The microbiota changes observed after EB-LFR exposure included reductions in *Gilliamella*, *Snodgrassella*, and *Lactobacillus*, accompanied by a transient increase in *Bifidobacterium* and the detection of *S. marcescens* and *E. hormaechei*. Similar patterns have been reported following exposure to antibiotics [35], neonicotinoids such as imidacloprid and thiamethoxam [7,8], and pyrethroids [9], which can alter epithelial integrity or immune signaling and thereby create ecological opportunities for opportunistic bacteria. The transient expansion of *Bifidobacterium* at 24 h resembles the stress-associated blooms described by Zheng et al. [32], which are generally interpreted as indicators of instability rather than improved gut function. The detection of *S. marcescens* is noteworthy, as this species has been associated with gut barrier disruption and systemic infection in bees [35]. Although mechanistic conclusions cannot be drawn from the present design, these patterns are consistent with the possibility that EB-LFR exposure may be associated with effects on host physiology that could influence the stability of the gut environment.

In contrast, RH-5849 was associated with a more moderate alteration of microbiota composition. Although no opportunistic taxa were detected, the pooled sample showed a broad reduction in core symbionts, including *Bifidobacterium*, *Gilliamella*, and *Lactobacillus*. This pattern aligns with the low acute toxicity observed in survival assays, where no statistically significant differences among treatments were detected, and mortality increased only gradually with dose. These taxa have been associated with carbohydrate metabolism, detoxification, and immune modulation in previous studies, and their reduction may indicate potential implications for host resilience over longer time scales, even in the absence of acute mortality [36]. Similar reductions in beneficial taxa have been reported following exposure to sublethal doses of neonicotinoids [37,39,40] and fungicides [50], and may reflect changes in host physiology that could influence microbial colonization. Although ecdysone agonists are generally considered reduced-risk insecticides with limited effects on non-target arthropods [51,52], our results suggest that they may still influence gut microbiota composition, highlighting a dimension of sublethal effects not captured by LD_50_-based assessments.

The temporal patterns observed after EB-LFR exposure provide tentative insight into microbiota dynamics. The gut community showed clear changes at 24 h, followed by partial recovery of *Gilliamella* and *Snodgrassella* at 48 h. This pattern could reflect the selective survival of bees with more resilient microbiota, the re-establishment of microbial niches, or competitive interactions among taxa. However, the persistence of *E. hormaechei* at 48 h suggests that recovery was incomplete. These observations are consistent with previous studies indicating that the bee gut microbiota can recover from moderate perturbation but may not fully re-establish homeostasis following stronger chemical stress [31,32]. Because the present study used pooled samples, these temporal patterns should be interpreted cautiously but may serve as a basis for future hypotheses.

Changes in gut microbiota composition may have implications for colony-level health. Core symbionts support nutrient assimilation, detoxification, and immune priming, and their depletion has been associated with reduced foraging efficiency, impaired brood care, and increased susceptibility to pathogens such as *Nosema* and *Serratia* [28,33]. The detection of opportunistic taxa following EB-LFR exposure may further increase vulnerability under conditions of nutritional stress or pathogen pressure. Thus, even when acute mortality is limited, alterations in gut microbiota composition may contribute to reduced colony resilience over time.

Several limitations of this study highlight directions for future research. Experiments were conducted under controlled laboratory conditions, which do not fully reflect the complexity of field environments where bees encounter chronic low-dose exposure, mixtures of agrochemicals, and fluctuating environmental stressors. A solvent-only control was not included for RH-5849 treatments, in which acetone was used as a carrier solvent; therefore, potential solvent-related effects cannot be fully excluded. In addition, because RH-5849 exhibited low toxicity and mortality differences among dose groups were not statistically significant at 24–48 h, the resulting LD_50_ estimate reflects a shallow dose–response relationship and should be interpreted with caution, although it remains suitable for toxicity classification. However, the comparative design still allows preliminary identification of treatment-associated microbiota patterns. Bees were obtained from a limited number of colonies, and because gut microbiota composition varies across colonies and seasons [24], broader sampling would strengthen generality. In addition, the microbiota analysis was based on a single pooled sample per treatment and time point, precluding statistical inference. Future studies incorporating biological replication at both individual and colony levels will be essential to validate the patterns observed here. Specific taxa (*S. marcescens* and *E. hormaechei*) were not independently validated and require future confirmation using targeted approaches (e.g., qPCR). The study focused on early responses (24–48 h), and longer-term effects on microbiota stability, pathogen susceptibility, and colony performance remain to be determined. Finally, 16S rRNA gene sequencing provides taxonomic resolution but limited functional insight. Integrating metagenomic, metatranscriptomic, or metabolomic approaches would help clarify the functional consequences of pesticide-associated microbiota changes.

Taken together, these exploratory results are consistent with differences in microbiota responses under exposure to insecticides with contrasting toxicity profiles. High-toxicity compounds were associated with reductions in core symbionts and the detection of opportunistic taxa, whereas low-toxicity compounds were associated with broader depletion of beneficial bacteria without apparent pathogen emergence. Although exploratory, these findings support the inclusion of gut microbiota endpoints in pesticide risk assessment and emphasize that compounds classified as reduced-risk may still exert biologically relevant sublethal effects not captured by conventional toxicity metrics.

## Data Availability

The raw 16S rDNA amplicon sequencing data generated in this study are available in the NCBI Sequence Read Archive (SRA) under accession number SRR37482898. The original contributions presented in this study are included in the article. Further inquiries can be directed to the corresponding authors.

## Abbreviations

The following abbreviations are used in this manuscript:

EB-LFR	Emamectin benzoate-lufenuron
LD_50_	Median lethal dose
a.i.	Active ingredient
DNA	Deoxyribonucleic acid
rRNA	Ribosomal ribonucleic acid
OTU	Operational taxonomic unit
ASV	Amplicon sequence variant
PCR	Polymerase chain reaction
SEM	Standard error of the mean
CI	Confidence interval
SRA	Sequence Read Archive
QIIME2	Quantitative Insights Into Microbial Ecology 2
ANOVA	Analysis of variance
